# Supplementary material for: Agricultural subsidies and global greenhouse gas emissions
Source: Nat Commun. 2021 May 10;12:2601. doi: 10.1038/s41467-021-22703-1 (PMC8110782; doi:10.1038/s41467-021-22703-1)
Supplement: Supplementary file 1 — Supplementary Information [file 41467_2021_22703_MOESM1_ESM.pdf]

Agricultural subsidies and global greenhouse gas emissions

David Laborde[1], Abdullah Mamun[1], Will Martin[1], Valeria Piñeiro[1], Rob Vos*[1]

[1] *International Food Policy Research Institute (IFPRI)*, 1201 I Street N.W., Washington D.C. 20007, USA

Supplementary Table 1:

Changes in Output Following Removal of Coupled Subsidies and Border Measures (%)[a]

	Farm	*Beef*	*Dairy*	*Rice*	*Pork/Poultry*
Coupled Subsidies					
Australia	1.7	1.4	1.1	1.0	0.5
Brazil	0.3	0.4	0.0	-1.5	1.1
China	-1.1	-0.1	0.1	-1.6	-0.4
EU	-3.4	-3.5	-1.5	-1.4	-2.5
India	-1.7	-2.1	0.2	-2.9	-1.2
Indonesia	-0.3	-0.3	0.6	-0.4	0.1
Japan	-2.9	-3.2	-3.8	-0.4	-0.3
Mexico	-3.3	-7.0	0.0	1.0	-4.2
Russia	-1.6	-0.5	-3.5	-0.8	-2.3
USA	0.0	0.1	-0.2	1.0	0.7
Developed	-1.7	-1.1	-1.3	-0.3	-1.2
Developing	-0.5	-0.2	0.3	-1.0	-0.3
World	-0.9	-0.7	-0.6	-0.9	-0.6
Border Measures					
Australia	20.9	31.2	40.2	22.3	2.7
Brazil	11.1	18.1	-1.1	-2.9	19.4
China	-3.6	-3.6	-39.6	-1.1	-0.1
EU	-1.3	-12.8	7.1	-20.1	-2.1
India	2.4	32.0	3.5	6.2	0.7
Indonesia	-5.2	-24.2	-0.8	-9.0	-5.0
Japan	-21.8	-32.6	-64.7	-4.4	-22.7
Mexico	-2.2	5.0	-1.7	-2.4	-10.3
Russia	-8.8	-11.7	-14.1	-11.5	-10.6
USA	4.9	3.8	8.7	29.6	4.8
Developed	-0.6	-3.0	1.4	-3.3	-1.3
Developing	0.1	2.7	-3.5	-0.2	1.3
World	-0.1	-0.2	-0.9	-0.4	0.3

Source: MIRAGRODEP simulations. See Ref. [Error! Reference source not found.] for further detail.

Note: Please note that the results in this table are shown as "counterfactuals", that is, what output would be in the absence of support measures relative to the existing situation. A negative value indicates therefore that existing support helps increase agricultural output.
